# Endotracheal aspirates contain a limited number of lower respiratory tract immune cells

**DOI:** 10.1186/s13054-020-03432-1

**Published:** 2021-01-06

**Authors:** Marika Orlov, Eric D. Morrell, Victoria Dmyterko, Jessica A. Hamerman, Mark M. Wurfel, Carmen Mikacenic

**Affiliations:** 1 Department of Veterans Affairs, Puget Sound, Hospitalist and Specialty Medicine, Seattle, WA 98108 USA; 2 Division of Pulmonary, Critical Care, and Sleep Medicine, Harborview Medical Center, University of Washington, Seattle, WA 98104 USA; 3 Benaroya Research Institute, Seattle, WA 98101 USA

To the editor:

Use of bronchoscopies for research during the COVID-19 pandemic has been limited due to risk of aerosol exposure and need to preserve PPE. Endotracheal aspirates (ETAs) have been used for research as they are easily obtained via a simple, non-aerosol generating procedure without need for extra PPE [1, 2]. However, there is a paucity of information regarding whether ETAs are a reasonable surrogate for bronchoalveolar lavage (BAL) to study lung specific immune responses in critically ill patients. The purpose of this study was to compare the immune cell populations detectable in ETA versus BAL using flow cytometry to evaluate the potential utility of ETAs for research.

We enrolled critically ill, non-COVID patients (*n* = 12) with suspected bacterial pneumonia on mechanical ventilation undergoing bronchoscopy with BAL, approved by the University of Washington Human Subjects Committee under a waiver of consent. Immediately before bronchoscopy, ETA was collected into a sputum trap via the in-line suction catheter passed through the ET tube to maximal depth. BALF was processed as described previously [3]. ETA was mixed with equal volume 0.1% DTT, incubated on ice for 15 min, and strained through a 70 µM cell strainer [4]. Cells were pelleted by centrifugation, washed in PBS, and cryopreserved. Later, cells were thawed and stained with a live/dead cell marker (eFluor780, eBiosciences), washed with PBS, and stained for 30 min with antibodies to the following extracellular markers: (eBiosciences) anti-CD45-FITC, anti-CD3-BV510, anti-CD4-BV421, anti-CD8-PE-Cy7, anti-CD14-PE, anti-CD206-PerCP-Cy5.5, and anti-CD20-APC. ETA and BALF cell proportions were measured by manual inspection of cytospins prior to cryopreservation (*n* = 8). Wilcoxon signed-rank test was used to compare percent populations of cells across the two groups, and Spearman’s rank-order test was used to identify correlations between ETA and BALF.

A majority of the patients were male (10/12, 83%), white (9/12, 75%), and average age was 54 years (range 30–72). Bronchoscopies were performed an average of 4.75 days post-intubation (range 1–11 days), and pneumonia was diagnosed by quantitative BAL culture in 50% of the samples (6/12). Manual inspection of cytospins demonstrated low percentages of neutrophils (ETA and BAL: 6% of all cells) and epithelial cells (ETA: 1% of all cells, BAL: 0%). Flow cytometric quantification of BAL showed CD206+ alveolar macrophages (36% of CD45+ cells, Table 1) and T- and B-lymphocytes (32% of CD45+ cells) to be the most abundant cell types. In contrast, the predominant cell type in ETA was CD14+ monocytes (65% of CD45+ cells). Despite differences in abundance by fluid type, we did observe moderate to high correlation in proportions for alveolar macrophage (*r* = 0.643, *p* = 0.028), CD4+ (*r* = 0.848, *p* = 0.001), CD8+ (*r* = 0.692, *p* = 0.016), and CD20+ lymphocytes (*r* = 0.587, *p* = 0.049, Fig. 1). Percentages of monocytes and total lymphocytes were not significantly correlated between the two samples.

**Table 1:** Immune cells found in bronchoalveolar lavage fluid and endotracheal aspirates

	BALF (%, SD)	ETA (%, SD)	*p* value (% BALF vs % ETA)	Spearman coefficient	*p* value (correlation)
Monocytes (CD45+CD14+)	26, +/− 22	65, +/− 11	*p* = 0.001	*r* = 0.350	*p* = 0.266
Alveolar macrophages (CD45+CD206+)	36, +/− 29	12, +/− 11	*p* = 0.005	*r* = 0.643	**p** = **0.028**
Lymphocytes (CD45+ , forward/side scatter)	32, +/− 14	15, +/− 11	*p* = 0.001	*r* = 0.580	*p* = 0.052
B Cells (CD45+ CD20+)	2, +/− 1.4	7, +/− 8	*p* = 0.030	*r* = 0.587	**p** = **0.049**
T Cells (CD45+ CD3+)	78, +/− 15	72, +/− 13	*p* = 0.176	*r* = 0.238	*p* = 0.457
CD4+T cells (CD45+CD3+CD4+)	55, +/− 23	65, +/− 18	*p* = 0.064	*r* = 0.848	**p** = **0.001**
CD8+cells (CD45+CD3+CD8+)	40, +/− 22	31, +/− 18	*p* = 0.077	*r* = 0.692	**p** = **0.016**
Unclassified CD45+cells	6, +/− 7	8, +/− 6			

The bold represents correlations that are significant with a *p* value < 0.05

Comparison of immune cell populations in bronchoalveolar fluid (BALF) versus endotracheal aspirates (ETA), *p* value was calculated using Wilcoxon signed-rank test. Correlation of immune cells between BALF and ETA was calculated using Spearman’s coefficient

**Fig. 1:**
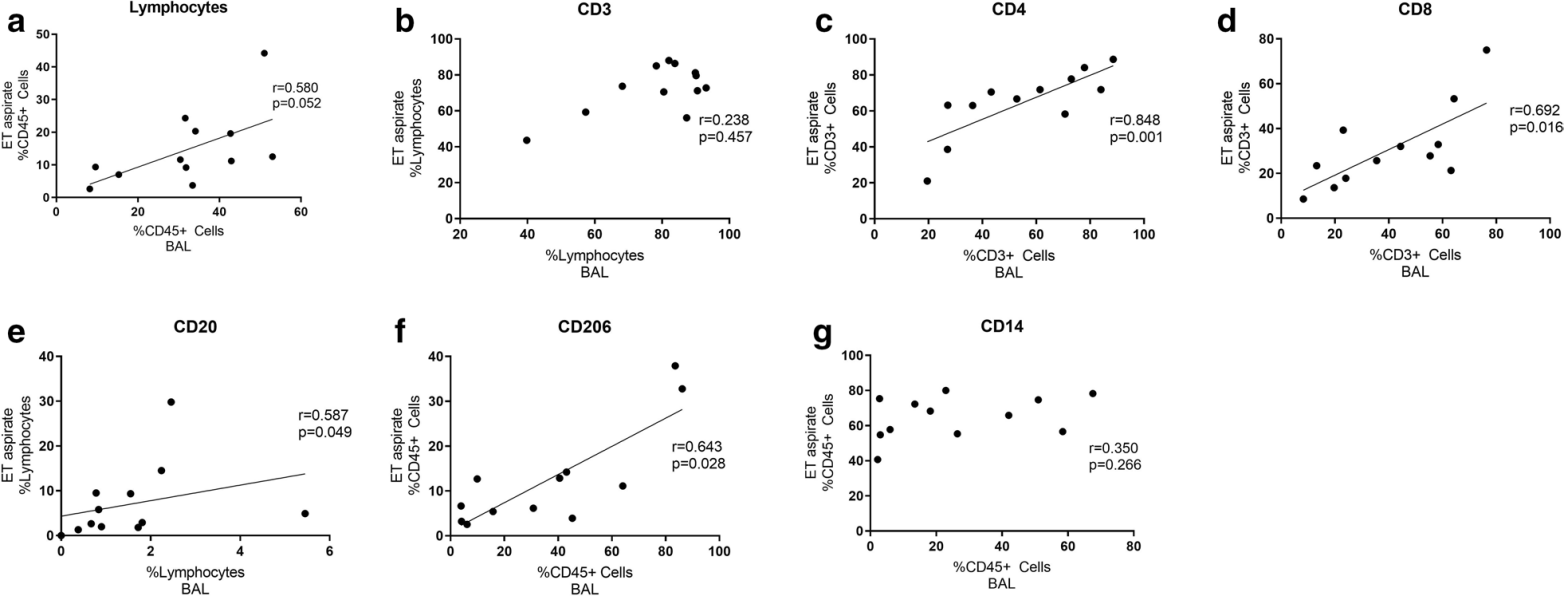
Correlation of immune cell populations between ET Aspirates and BAL Fluid. Spearman’s rank-order test was used to determine correlation coefficients and significance. Best fit line added in correlations that reach/approach significance

We show that there are immune cell subsets present in ETA in critically ill patients and that these subsets are distinct from what is found in BALF. Also, we were able to recover alveolar macrophages from ETA, and while the percentage of alveolar macrophages between paired ETA and BALF samples are different, they do correlate, suggesting that ETA may be useful to study specific cell populations. One limitation of this study is that the cells were passed through a 70 µM filter, filtering out clumps of neutrophils and NETs, likely explaining the low numbers of neutrophils in these samples. In conclusion, these findings suggest that ETA samples contain populations of immune cells from the lower respiratory tract of critically ill patients with respiratory failure, but that these samples are not necessarily a surrogate for BALF in research studies. Care should therefore be used in interpreting studies in critically ill patients using ETA.

## Data Availability

Data can be submitted as an excel spreadsheet at the reviewer’s request.
